# Supplementary material for: The interactome of intact mitochondria by cross-linking mass spectrometry provides evidence for coexisting respiratory supercomplexes
Source: Mol Cell Proteomics. 2017 Dec 8;17(2):216–32. doi: 10.1074/mcp.RA117.000470 (PMC5795388; doi:10.1074/mcp.RA117.000470)
Supplement: Supplemental Data [file supp_RA117.000470_133922_0_supp_23978_fzffwf.zip › spectra_annotation/mito_DR_spectra_annotation/338-1-12-1-3-1.pdf]

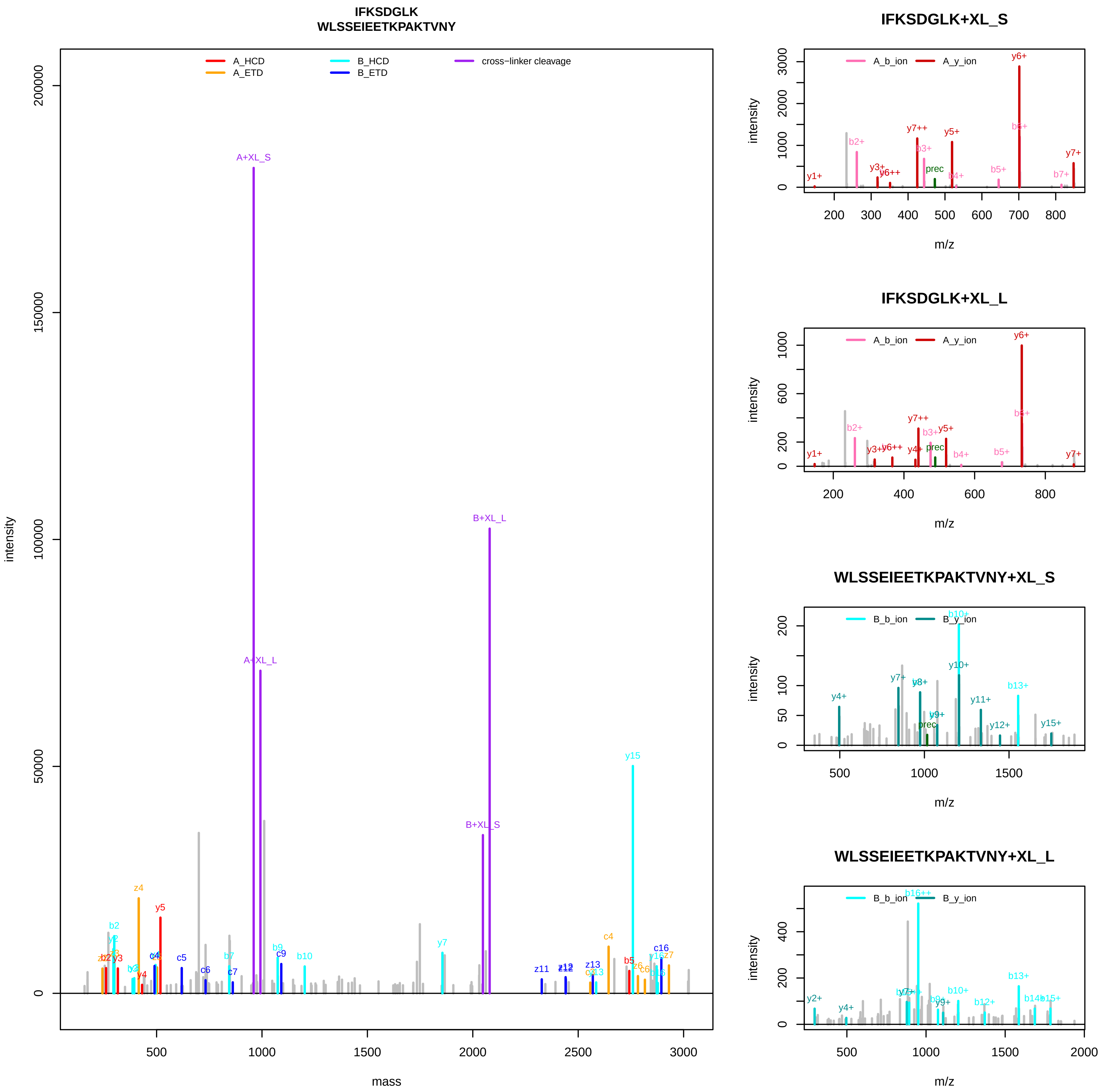
IFKSDGLK
WLSSEIEETKPAKTVNY
A_HCD
A_ETD
B_HCD
B_ETD
cross−linker cleavage
A+XL_S
A+XL_L
B+XL_L
B+XL_S
y15
y7
z4
y5
b2
c4
c5
c6
c7
b7
b9
c9
b10
z11
z12
z13
c4
b5
c16
z7
intensity
mass
0
50000
100000
150000
200000
500
1000
1500
2000
2500
3000
IFKSDGLK+XL_S
A_b_ion
A_y_ion
y6+
y7++
y5+
b2+
b6+
b3+
y3+
y6++
prec
b5+
b4+
y1+
y7+
b7+
intensity
m/z
IFKSDGLK+XL_L
WLSSEIEETKPAKTVNY+XL_S
B_b_ion
B_y_ion
b10+
y10+
y7+
y8+
y4+
y11+
b13+
y9+
y12+
y15+
prec
WLSSEIEETKPAKTVNY+XL_L
b16++
b13+
b10+
b12+
y2+
y4+
y9+
b14+
b15+
